# Pathologists’ first opinions on barriers and facilitators of computational pathology adoption in oncological pathology: an international study

**DOI:** 10.1038/s41388-023-02797-1

**Published:** 2023-08-16

**Authors:** Julie E. M. Swillens, Iris D. Nagtegaal, Sam Engels, Alessandro Lugli, Rosella P. M. G. Hermens, Jeroen A. W. M. van der Laak

**Affiliations:** 1 Scientific Center for Quality of Healthcare (IQ Healthcare), Radboud Institute for Health Sciences (RIHS), Radboud University Medical Centre, Nijmegen, The Netherlands; 2 Department of Pathology, Radboud Institute for Molecular Life Sciences (RIMLS), Radboud University Medical Centre, Nijmegen, The Netherlands; 3 Institute of Pathology, University of Bern, Bern, Switzerland; 4 Center for Medical Image Science and Visualization, Linköping University, Linköping, Sweden

**Keywords:** Cancer, Biological techniques

## Abstract

Computational pathology (CPath) algorithms detect, segment or classify cancer in whole slide images, approaching or even exceeding the accuracy of pathologists. Challenges have to be overcome before these algorithms can be used in practice. We therefore aim to explore international perspectives on the future role of CPath in oncological pathology by focusing on opinions and first experiences regarding barriers and facilitators. We conducted an international explorative eSurvey and semi-structured interviews with pathologists utilizing an implementation framework to classify potential influencing factors. The eSurvey results showed remarkable variation in opinions regarding attitude, understandability and validation of CPath. Interview results showed that barriers focused on the quality of available evidence, while most facilitators concerned strengths of CPath. A lack of consensus was present for multiple factors, such as the determination of sufficient validation using CPath, the preferred function of CPath within the digital workflow and the timing of CPath introduction in pathology education. The diversity in opinions illustrates variety in influencing factors in CPath adoption. A next step would be to quantitatively determine important factors for adoption and initiate validation studies. Both should include clear case descriptions and be conducted among a more homogenous panel of pathologists based on sub specialization.

## Introduction

Over the past decade, advances in scanning and storage hardware have resulted in widespread use of whole slide images (WSI) in pathology, often referred to as ‘digital pathology’. Digital pathology opens the door for applying machine learning techniques capable of extracting diagnostic information from scanned slides. An example can be seen supporting oncological diagnostics [1]. The most widely used machine learning techniques for WSI are convolutional neural networks (CNN), which are a type of deep learning models that are extremely powerful for analyzing image data [2–5]. Successfully developed CNN can automatically detect, segment, or classify cancer in WSI. Their capabilities approach or even exceed the accuracy of pathologists for specific tasks primarily within oncological pathology [3, 6].

Using deep learning for WSI (computational pathology; CPath) can increase efficiency by potentially reducing pathologists’ workload and automating repetitive task of low complexity such as screening for metastases within lymph nodes of breast cancer patients [4]. It may also be helpful in evaluating biomarkers that are hampered by significant inter-observer variability to increase accuracy, speed and objectivity of diagnoses [1, 3, 6], thereby facilitating accurate treatment decisions. Examples are Gleason grading of prostate cancer [2] and the detection of tumor buds within early colorectal tumors [7]. In addition, CPath can also potentially yield new diagnostic clues which have not been recognized by pathologists before [8].

Despite the promising results of CPath, several challenges have to be explored and addressed before it can be used in clinical practice: 1) building trust in using of CPath within medical practice (presuming deep learning models are represented as black boxes); 2) developing robust and trustworthy CPath trained with high-quality data from various sources to increase generalizability and prevent selection bias; 3) conducting large-scale (preferably prospective) peer-reviewed validation studies showing impact on patient care; 4) deciding on how to incorporate CPath into daily routine practice, including the assignment of responsibility; 5) finding solutions to ethical concerns; 6) certifying CPath to acquire a legal basis [2, 3, 6, 9–12].

Implementation is often only considered after an innovation is already widely available in clinical practice. However, concerning the future use and of CPath applications in clinical practice, early involvement of potential end-users is critical for gaining wider clinical usage and tailoring future implementation strategies to the needs of the potential end-users [13]. Current literature entails many influencing factors from a CPath developer perspective, but perspectives on CPath adoption from the end-users are limited [14]. As challenges of CPath clinical use are present at a global level, multiple countries should be involved in this explorative process. Therefore, the objective of this study is to explore international perspectives on the future role of CPath in clinical practice by focusing on opinions and first experiences regarding barriers and facilitators. These opinions and first experiences will inform the development of validation studies, implementation trajectories and communication activities for creating widespread stakeholder acceptance.

## Results

### Literature study

We found 14 review studies in total describing barriers and facilitators for CPath clinical use [8, 15–27]. Strengths of CPath use were by far mostly mentioned in these studies. Other common topics extracted were barriers regarding quality of evidence supporting CPath outcomes and potential lack of trust or acceptance of AI systems by pathologists. Facilitators were clarification on AI training and the need for completely digitized pathology workflows.

### eSurvey

**Fig. 1:**
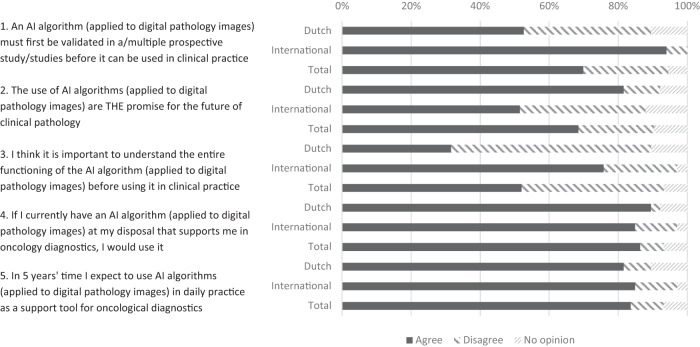
Answers to statements in eSurvey.

The eSurvey yielded 70 responses in total, including 38 pathologists working in the Netherlands and 32 working abroad. Figure 1 shows the replies to the statements, in total and disaggregated for the two subgroups. Respondents’ characteristics are shown in Table 1. Dutch respondents were represented by more non-academics than with international respondents.

**Table 1:** eSurvey respondents’ and interview participants’ characteristics.

	eSurvey Dutch (*n* = 38)		eSurvey International (*n* = 32)		eSurvey Total (*n* = 70)		Interviewees (*n* = 16)	
*Characteristics*								
Mean age (years)	44		45		44		46	
	*n*	%	*n*	%	*n*	%	*n*	%
**Gender**								
*Male*	15	40%	19	59%	34	49%	9	56%
*Female*	21	55%	13	41%	34	49%	7	41%
*Unknown*	2	5%	0	0%	2	3%		
**Function**								
*Pathology resident*	4	11%	5	16%	9	13%	1	6%^a^
*Pathologist (generalist)*	9	24%	7	22%	16	23%	3	19%
*Pathologist (specialist)*	15	39%	16	50%	31	44%	12	75%
*Unknown*	10	26%	4	12%	14	20%		
**Involvement CPath development**								
*Pathologist and involved in CPath development*	6	16%	6	19%	12	17%	8	50%
**Type of hospital**								
*Pathologist (non-academic)*	16	42%	2	6%	18	26%	9	56%
*Pathologist (academic)*	10	26%	12	38%	22	31%	7	41%
*Unknown*	12	32%	18	56%	30	43%		
**Region**								
*The Netherlands*	38	100%			38	54%	8	50%
*Europe*			10	31%	10	14%	4	25%
*United Kingdom*			2	6%	2	3%		
*Northern America*			1	3%	1	2%	2	13%
*Africa*							1	6%
*Asia*							1	6%
*Unknown*			19	60%	19	27%		

^a^One interview participant was selected on the basis of being a pathology resident, but at the time of the interview, the participant had become a pathologist.

Overall, most respondents had a positive attitude towards CPath use in clinical practice: 61 out of 70 (87%) would currently start using CPath algorithms when available as a support tool in oncology diagnostics. A similar percentage of respondents (83%, *n* = 58) expected to be using CPATH algorithms in clinical practice in 5 years from now. Sixty-seven percent (*n* = 47) of respondents perceived CPath as the future promise in clinical pathology, with Dutch pathologists having a more positive view (82%, *n* = 31 vs. 50%, *n* = 16). In line with this point, almost all international respondents demanded prospective validations studies (94%, *n* = 30), whereas only half of Dutch respondents (53%, *n* = 20) needed this before clinical adoption. Similarly, fewer Dutch pathologists required a full functional explanation of the CPath algorithm (32%, *n* = 12 vs. 78%, *n* = 25).

### Semi-structured interviews

In total, we interviewed 15 pathologists and 1 pathology resident, of which eight were working in the Netherlands. Average years of experience was 14 (range 1–30). Diverse areas of focus were represented in the interview study. Common areas such as breast cancer and gastro enterology were included, but also uncommon ones such as pediatric and endocrine diagnostics. The interviewees’ characteristics can be found in Table 1.

We found opinions and first experiences regarding 65 barriers and 130 facilitators for implementing CPath algorithms in histopathology, of which 29 barriers and 72 facilitators were mentioned in at least two interviews (Tables 2 and 3). These influencing factors are illustrated with quotes (Table 4). Some quotes were translated from Dutch to English.

**Table 2:** Barriers for CPath use in clinical practice mentioned in ≥2 interviews (*n* = 29).

Innovation factors	No of interviews (*n* = 16)	Individual health professional factors	No of interviews (*n* = 16)	Incentives and resources	No of interviews (*n* = 16)	Social, political and legal factors	No of interviews (*n* = 16)
*Compatibility*		*Agreement with recommendation*		*Availability of necessary resources*		*Legislation*	
• Vulnerability of CPath depending on quality of previous steps workflow	3	• Critical attitude towards CPath clinical use	9	• Insufficient staining quality	2	• Liability position/responsibility of pathologists regarding CPath output	7
				• Unavailability of digital workflow	2	• Unclearness regarding liability in case of error CPath	3
						• Unawareness regarding applicable regulations CPath use	3
*Feasibility*		*Awareness and familiarity*		*Financial (dis)incentives*			
• More difficult to develop CPath for rare cancer types	2	• Perception of CPath being a “black-box”	6	• Tremendous financial investment	3		
• Time associated with CPath implementation process	2	• Limited knowledge regarding CPath	5	• Relative small budget pathology departments	2		
• Effort to guide CPath before and after tissue analysis	2	• Lack of experience regarding CPath	4				
• Hard to imagine specific leading role of CPath within workflow	2						
*Quality of evidence*		*Expected outcome*					
• Questionable reliability of CPath	4	• Potential clinical impact of rather minor deviations CPath (e.g. Gleason grading)	2				
• Problems regarding determination ground truth	4	• Potential clinical impact of accepted error margins CPath	2				
• Questionable improvement in clinical practice due to CPath	3						
• Hard to determine cause-effect relationship between CPath use and clinical outcomes	4						
• Time associated with prospective clinical trials	5						
• Uncertainty regarding CPath validation on local level	5						
*Source of the recommendation*		*Domain knowledge*					
• Potential conflict of interest CPath suppliers	2	• Loss of domain knowledge due to CPath	2				
		*Emotions*					
		• Fear of losing job	4				
		*Skills needed to adhere*					
		• Loss of diagnostic skills	7				
		• Becoming too reliant on CPath	4

**Table 3:** Facilitators for CPath use in clinical practice mentioned in ≥2 interviews (*n* = 72).

Innovation factors	No of interviews (*n* = 16)	Individual health professional factors	No of interviews (*n* = 16)	Professional interactions	No of interviews (*n* = 16)
*Accessibility intervention*		*Agreement with recommendation*		*Communication & influence*	
**•** Availability of stand-alone CPath	2	**•** Positive attitude	14	• Encouragement by clinicians	7
• Availability open source CPath applications	3	• Outcome control of CPath remains necessary	6	• Encouragement by pathologist colleagues	2
*Compatibility*		*Awareness and familiarity*		*Team processes*	
• (Easy) integration of CPath in IT structures	6	• CPath use in clinical practice	2	• Align CPath use with clinicians	2
• CPath analysis on background	4	• Involved in CPath development	6	• Clinicians trust pathologists in use/ not use CPath	4
• CPath being able to sort cases on urgency within workflow	3	• Gaining basic understanding of CPath	3	• Including use of CPath in pathology reporting	10
• First tissue analysis by CPath, next pathologist (supportive application)	9	• Having basic understanding of CPath	11		
• First tissue analysis by pathologist, next CPath • (leading application)	9	• Gaining trust in CPath step by step	8		
*Feasibility*		*Intention and motivation*			
• Usefulness depends on speed and user-friendliness CPath	4	• Having appointments with suppliers CPath	2		
• Leading role “standard” work	6	• Intention to use CPath for several applications^b^	11		
• Complex diagnostics	2	• CPath use in near future	4		
*Quality of evidence*		*Skills needed to adhere*			
• Including clinical outcomes in CPath development	2	• Data integration task when CPath is used	7		
• Variety of data needed in CPath development	2				
• Validation of CPath	9				
• Proven reliability CPath	7				
• Specific validation with clinical outcomes	10				
• Validation non-inferiority studies	3				
• Validation per laboratory	6				
• Prospective validation studies needed	5				
• Retrospective validation studies sufficient	4				
• Studies on time savings	2				
*Source of the recommendation*					
• Reliable supplier CPath, including data usage	5				
• Validation by supplier	4				
• Ongoing development CPath by supplier	2				
• No preferences supplier	4				
*Strength of the recommendation*					
• Advantage of time-savings	12				
• Decreasing repetitive tasks	4				
• Improving workflow efficiency	5				
• Improving diagnosis quality/accuracy	9				
• Better definition of currently known prognostic factors	5				
• Improving standardized diagnostic outcome CPath	10				
• Improving detection due to narrowing analysis area	5				
• Lowering workload	2				
• More comprehensive CPath	2				
• Finding new prognostic factors	7				
• Improving treatment choices for patients	2				

Incentives and resources	No of interviews (*n* = 16)	Capacity for organizational change	No of interviews (*n* = 16)	Social, political and legal factors	No of interviews (*n* = 16)
*Availability of necessary resources*		*Mandate, authority, accountability*		*Legislation*	
• Availability of digital workflow	2	• Updating CPath centrally	2	• FDA^c^ approved CPath	2
• One supplier for entire digital workflow including CPath	2	• Central implementation pathology association	7	• Global regulation CPath clinical use	2
• Available CPath applications	3			• Autonomous decision pathologist CPath use because accountability	6
*Continuing education system*		*Monitoring and feedback*			
• CPath in pathology resident education	13	• Providing feedback to supplier of CPath	2		
• More explainable pathology due to CPath	2	• Central monitoring system	3		
• Learning pathology analysis before CPath use in resident education	4	• Prospective monitoring CPath clinical use	9		
• Anticipating in labor market because of use of CPath	3				
• CPath as part of continuing pathology education	2				
*Information system*		*Regulations, rules, policies*			
• Automatic fill-in SSR^a^	5	• Policy of pathology association regarding CPath use	3		
• Connecting CPath with other information systems	4				
• Enable to assign CPath to case manually	2				
*Quality assurance and patient safety systems*					
• Upfront quality assurance	10				

^a^Standardized structured reporting.

^b^Applications for lymph node screening, Gleason grading, and quantifying tasks (mitosis or Ki67 counting).

^c^U.S. Food and Drug Administration.

**Table 4:** Illustrative quotes about barriers and facilitators of CPath clinical use.

### Innovation factors – CPath algorithms

Most barriers regarding CPath algorithms related to quality of evidence: Some interviewees doubted the reliability of CPath. One of the reasons shared, is using pathologists’ expertize which is subject to inter-observer variability as the reference standard in supervised learning for CPath development. Concerns were also expressed regarding the actual impact of CPath use in clinical practice and its prospective and local validation. Regarding feasibility, since a large amount of data is required to train CPath algorithms, pathologists expect it will be challenging to develop CPath algorithms for rare cancer types. Pathologists who already used CPath algorithms mentioned the additional effort to manually select an area before applying the mitosis counting CPath algorithm and correcting the CPath output after tissue analysis as barriers for implementing CPath in daily practice. Also effort is needed to implement CPath in daily practice. Another barrier related to CPath’s compatibility is the quality of CPath algorithms being dependent on the quality of the steps in the workflow taken before slide digitization. Another barrier may be CPath being supplied by commercial parties with potential conflicts of interest in scientific publications supporting the CPath algorithms and lacking knowledge regarding the specific medical context in which CPath algorithms will be used.

In addition to these barriers, many facilitators were mentioned. Many strengths of using CPath in clinical practice were recognized. Clinical use of CPath will ultimately result in decreasing workload, better treatment choices, finding new prognostic factors and developing more comprehensive CPath algorithms. Corresponding to quality of evidence, proper development and proven reliability by internal and external validation of CPath algorithms were mentioned as facilitators. There is disagreement regarding the methodology required to determine clinical benefit and whether retrospective or non-inferiority studies sufficient or will only prospective clinical trials be valid. Concerning compatibility, interviewees shared different intents for using CPath within their workflow and having a leading or supportive function in the diagnostic process. The preferred function was mainly argued by the type of task and perceived reliability of CPath. In general, CPath should be integrated within existing digital workflows while also being able to run in the background. Regarding feasibility, interviewees asked for fast-analyzing user-friendly CPath for both “standard” and more complex diagnostics. Some interviewees additionally argued the necessity of CPath outcome control. Few interviewees argued that accessibility of CPath should not be limited to the CPath product range of scanner suppliers, while others asked for full open-source CPath algorithms. Sufficient validation, safe data use and ongoing development by reliable CPath suppliers were deemed necessary.

### Individual health professional factors – pathologists and pathology residents

Critical attitudes regarding CPath among potential end-users were present, illustrated by the statement that the additional value of clinical use should first be demonstrated sufficiently, especially before providing a leading function for CPath within the pathology diagnostic workflow. Critically assessing their own awareness and familiarity, many interviewees found that they lacked knowledge and experience with CPath and perceived its technique as a black-box. Even so, opinions differed on whether pathologists should understand the functioning of CPath. Concerns regarding expected outcome focused on the potential impact of rather minor deviations which could still have an impact on clinical outcome. Clinical introduction may also be hindered by the fact that only a small error margin will be acceptable to users for an entirely new technology. Concerns were raised about a loss of domain knowledge and skills within the field of pathology with users becoming too reliant on CPath. Emotions linked to the use of CPath were fear of job loss.

Despite these barriers, there was a positive attitude toward CPATH algorithms in general. To become more familiar and gain trust with CPath algorithms, a step-by-step approach was mostly suggested. Some were already involved in CPath research or were already using CPath in daily practice. Regarding intention and motivation, most interviewees intend to start using CPath for applications for quantifying tasks such as Gleason grading and lymph node screening. With CPath algorithms performing solely isolated tasks, interviewees foresaw processing and integrating a wide variety of data from different sources as a key skill when using CPath in clinical practice.

### Professional interactions – laboratory and multidisciplinary team

**Fig. 2:**
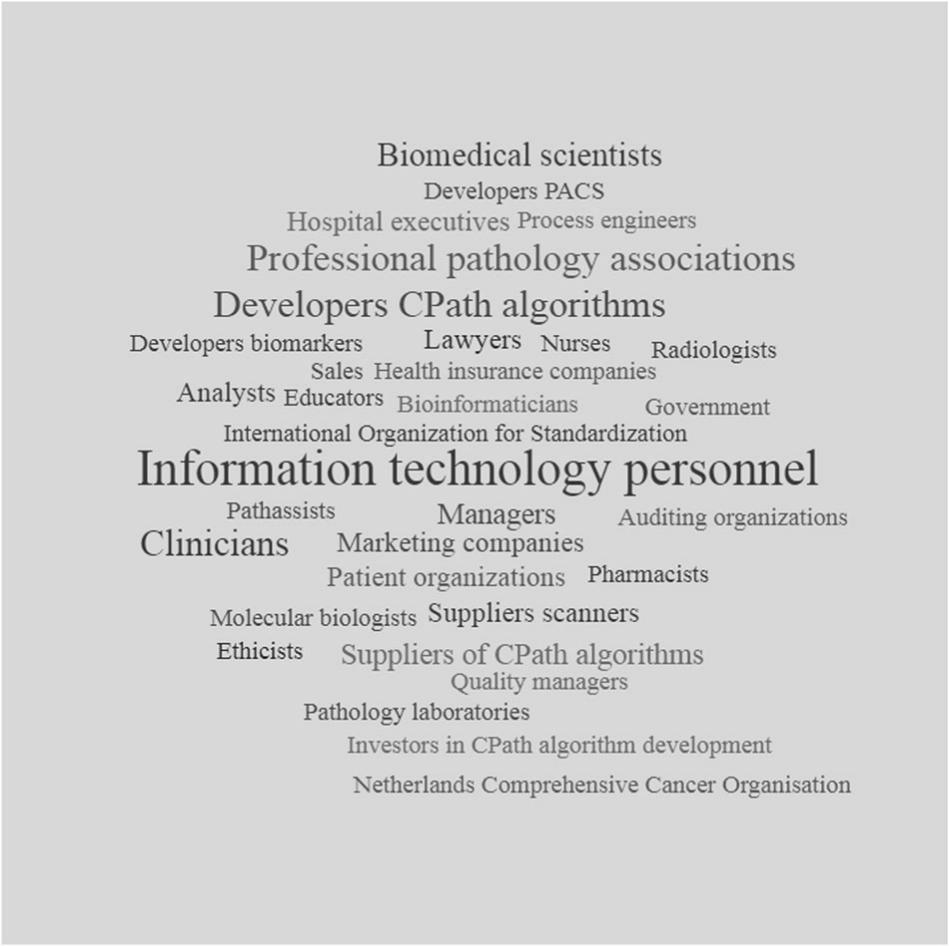
Wordle of stakeholders mentioned by interviewees.

Two facilitators were mentioned with regard to the social setting, namely that clinicians may encourage pathologists if CPath is arguably the better option to use and usage by pathologist-colleagues may lead to wider adoption. Concerning team processes, some interviewees thought clinicians should trust pathologists in considering clinical usage of CPath without consulting them. Others mentioned discussion of CPath use by pathologists with clinicians as a facilitator. Pathologists should inform clinicians by including information on CPath usage in pathology reports. A wide variety of stakeholders (*n* = 33) were deemed important by the interviewees as potentially having a role in CPath usage in clinical practice (Fig. 2). At both a local and national level, the most important stakeholders were information technology experts, professional pathology associations, auditing organization, clinicians and CPath developers.

### Incentives and resources – Hospital or external laboratory

Insufficient staining quality was seen as a potential barrier for CPath clinical use. Financial barriers include setting up a digital workflow to enable CPath use within the relatively small budgets of pathology departments.

Having one supplier for the entire digital workflow, including CPath, was seen as a facilitator. In some pathology laboratories with digital workflows, CPath applications were already available for quantifying tasks. Educational activities regarding CPath clinical use were mentioned as a facilitator. There was no consensus on the timing of CPath introduction in the training of residents. CPath applications need to be connected to other information systems, such as the laboratory management system (LMS), picture archiving and communication system (PACS), e.g. assigning CPath to cases manually. Furthermore, CPath should be able to automatically fill in templates used for pathology reporting. A quality certification was deemed necessary to guarantee quality assurance.

### Capacity for organizational change – Hospital or external laboratory

To determine capacity for organizational change, facilitators regarding mandate, authority and accountability concerning the central guidance of CPath use in clinical practice, by national pathology associations e.g. developing guidelines. In addition, CPath applications should be centrally updated to comply to updated versions of clinical guidelines. Prospective and central monitoring was reviewed as a facilitator, as was sending feedback on CPath clinical performance to the supplier.

### Social, political and legal factors – Healthcare regulation

Considering legal factors, the uncertainty about the liability position of pathologists, who are currently being responsible for their own output, and uncertainty in case of CPath error were considered barriers. This relates to the barrier of lacking awareness regarding applicable legislation for CPath clinical use. Therefore, a facilitator is the autonomical decision of pathologists to use CPath clinically without interference of a clinician. Having global regulations in place and U.S. Food and Drug Administration (FDA) approved CPath applications were other facilitators.

## Discussion

Our study provides an extensive overview of current opinions and first experiences regarding barriers and facilitators of CPath algorithm clinical use from an international perspective of direct users. Most barriers and facilitators determined by the interviews were categorized within the domain of the innovation itself and mainly concern the quality of evidence of CPath algorithms and their compatibility with current pathology laboratory workflows. The eSurvey study conducted prior to the interviews showed remarkable differences among Dutch and non-Dutch pathologists, particularly regarding their attitude and need to understand the entire functioning of CPath algorithms.

Our study shows that pathologists and pathology residents hold different opinions regarding important challenges in CPath clinical adoption, some of which are also presented by other research [6, 14, 28]. Moreover, these opinions may differ between countries and regions. A recent Delphi study showed a lack of consensus about the adoption of AI algorithms even amongst pathologists experienced in developing and evaluating CPath algorithms [14]. This, together with our results, stresses that many different aspects need to be addressed before interviews with end-users and further evaluations.

In the review of Van der Laak et al. [6], validation of CPath algorithms in pathology is stated as a current challenge, with different levels of validation being presented. Our study shows that both internal and external validation are deemed necessary among pathologists. However, various opinions were shared whether prospective validation should be performed before CPath algorithms can be used in clinical practice, thereby also taking into account the time and effort needed to perform these types of studies. Nagendran et al. [29] concluded that only a few randomized controlled trials have been performed on AI in medical imaging. For radiology specifically, Van Leeuwen et al. [30] assessed the efficacy of 100 CE marked AI algorithms and arrived at a similar conclusion, debating that the level of evidence should be associated with the intended use in clinical practice, distinguishing AI algorithms that are aimed at solely improving efficiency, diagnostic accuracy or also clinical outcomes. Future research should entail appropriate validation studies regarding the effectiveness of intended CPath algorithm use in clinical practice, as these findings can be included in clinical practice guidelines to guide pathologists on appropriate CPath algorithm clinical use.

Corresponding with qualitative findings of Chen et al. [31] among radiologists and radiographers, pathologists highlighted their ability as a medical professional to use AI algorithms to improve their diagnostic process in terms of both efficiency, accuracy and quality. However, in line with another study exploring perceptions of AI application use among healthcare professionals, pathologists also experienced a lack of knowledge regarding AI, sharing a need for training [32]. Despite perceiving CPath algorithms as black boxes, opinions varied whether pathologists should gain in-depth knowledge on the functioning of CPath before using it in clinical practice. More interest was shown for a step-by-step relation building approach, potentially facilitated by a real-world simulation digital environment. Several studies demand research into the interaction of humans with AI systems [10, 33]. Therefore, future research should focus on incorporating CPath into digital workflows and educational support which takes into account the differences in intended use and evidence regarding the interaction of humans with AI systems.

In line with other studies [6, 10] and also part of the action plan of the FDA [34] and post-market requirements of the CE-IVD [35], interviewees requested performance monitoring, assuring the safe and reliable clinical use of CPath algorithms and contributing to prospective evaluation: By periodically assessing patient outcomes, trends based on these outcomes can be compared to previous years and confidence intervals can be used to timely retrieve errors. However, such a data infrastructure aims for increased collaboration between regional, national and international pathology aligned associations and is not globally available.

Strength of our study is the inclusion of a diverse, international panel of pathologists and pathology residents to gather opinions and experiences regarding barriers and facilitators of a fast-developing innovation within oncology care, specifically pathology. In addition, by using an implementation science framework, a broad range of opinions and first experiences regarding influencing factors was identified. These can be used by researchers, clinicians and policy makers to determine CPath algorithm implementation readiness within their own context. A limitation of this study is the lack of recommendations of the use of CPath algorithms in clinical guidelines. The majority of the interviewees did not even have any experience with CPath algorithms. Therefore, their shared perspectives are mostly based on expectations instead of experiences. In addition, especially compared to radiology, pathology is still in the early phases of digitalization. A digital workflow using whole slide images instead of microscopes for diagnostics should be implemented first. Afterwards, CPath algorithms can be implemented to support diagnostics. Taking into account their knowledge and experience of other digital innovations within pathology and the time needed to develop implementation strategy elements, our study provides an interesting insight into the various opinions among pathologists regarding CPath implementation. These opinions can be used in the next steps toward clinical acceptance and implementation. A limitation may be our recruitment strategy, which is susceptible to selection bias, as we only included a small percentage of the total international pathology community. One of the most important distinctions between the full pathology population and our sample is the level of adoption of a digital pathology workflow, which varies between countries and hospital types. In the Netherlands, digital workflows are common in pathology laboratories, which may explain the more positive opinions on clinical use of CPath algorithms in general. Low rates of digitalization could especially be seen in international non-academic settings, which was represented poorly in this sample. However, this study aimed to explore barriers and facilitators among a diverse group of pathologists and showed important challenges among CPath algorithm development and validation from pathologists’ perspectives. These challenges should first be tackled before a wide scale implementation is considered. To be able to prioritize the most important factors, the results of our study should be quantified first among a representative group of international operating pathologists within a specific field of oncological pathology and other additional stakeholders. To overcome both limitations, in a next step international prospective validation studies could be conducted, using a hybrid design for testing both the effectiveness of the intervention itself (CPath algorithms) while simultaneously gathering quantitative information on implementation [36, 37].

## Conclusion

The extensive overview of barriers and facilitators associated with clinical adoption of CPath reveals a variety of opinions among end users and underlines the complexity of future CPath implementation in oncological pathology. Our results provide the basis for subsequent validation studies and implementation. Quantitative studies are necessary for prioritization, as well as well-defined use cases, with specific CPath algorithms and their target audience, to gain widespread acceptance of these new developments. Combining validation and implementation studies using a highly engaging hybrid format will be necessary to gain widespread stakeholder acceptance and keep up with the high speed developments within the field of computational pathology.

## Methods

### Study design

We carried out a narrative literature study to determine barriers and facilitators of the future clinical use of CPath algorithms. Results of this literature study were used to set up both an eSurvey and an interview guide. In the eSurvey, we explored the first reactions of using CPath in histopathology practice. Subsequently, we conducted online semi-structured interviews to more extensively explore pathologists’ perspectives on using CPath.

### Study population

**Fig. 3:**
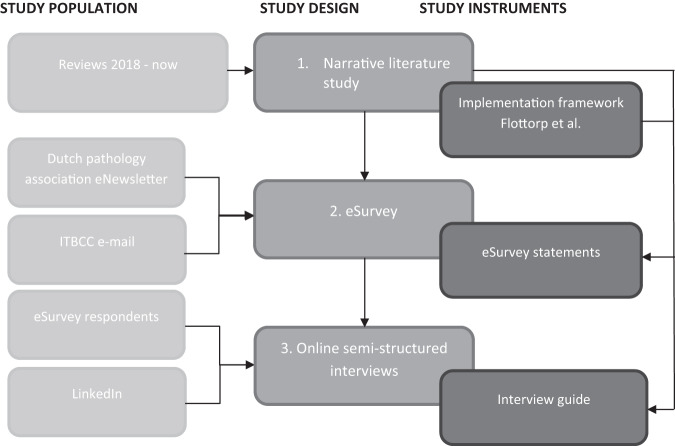
Flowchart including study population, study design and study instruments.

Aiming to include CPath end users in our explorative study, we recruited a wide variety of Dutch and international pathologists and pathology residents. We shared the eSurvey via two consecutive news items in the Dutch Pathology Association eNewsletter and through a directed e-mail message sent to members of the international tumor budding consortium (ITBCC) [38]. Respondents providing their e-mail addresses were considered candidates for the successive interview study. Based on their eSurvey results, pathologists and pathology residents with varying attitudes regarding CPath were selected for the interview study. Using LinkedIn, we requested additional respondents with a critical attitude towards CPath. The study design and related study population and study instruments is shown in Fig. 3.

### Data collection

We first conducted a quick literature scan in PUBMED, including key words, medical subject headings (MeSH) terms and synonyms for “pathology”, “algorithms” and “practice”. In addition, we excluded animal studies and included review studies and articles published after 2017. The search strategy is presented in Supplementary File 1. An eSurvey was established including five statements about CPATH, based on barriers and facilitators often mentioned in literature as well as questions regarding respondents’ age, sex, occupation (pathologist/resident), type of laboratory (academic/non-academic), involvement in artificial intelligence (AI) development, and request to optionally provide their e-mail address for participation in the consecutive interview study. The eSurvey statements are presented in Table 5.

**Table 5:** eSurvey statements.

1	An AI algorithm^a^ (applied to digital pathology images) must first be validated in a/multiple prospective study/studies before it can be used in clinical practice
2	The use of AI algorithms (applied to digital pathology images) are THE promise for the future of clinical pathology
3	I think it is important to understand the entire functioning of the AI algorithm (applied to digital pathology images) before using it in clinical practice
4	If I currently have an AI algorithm (applied to digital pathology images) at my disposal that supports me in oncology diagnostics, I would use it
5	In 5 years’ time I expect to use AI algorithms (applied to digital pathology images) in daily practice as a support tool for oncological diagnostics

^a^AI algorithms refers to the same concept as CPath algorithms.

An interview guide based on the literature scan was simultaneously developed (Supplementary File 2) regarding opinions and first experiences with CPath for identifying barriers and facilitators of CPATH clinical use. The opinions on factors influencing CPath usage found in 14 review articles were mapped onto the categories of the domains of the implementation theory framework of Flottorp et al. [39]. In addition, questions based on subdomains of Flottorp et al. [39] not yet mentioned in literature were added to the interview guide. The interview guide mainly consisted of questions and statements aiming to encourage participants to actively think about future challenges of CPath. The interview guide was first tested among the researchers themselves (JS and SE) and finally with a pathology resident actively conducting CPath research.

**Fig. 4:**
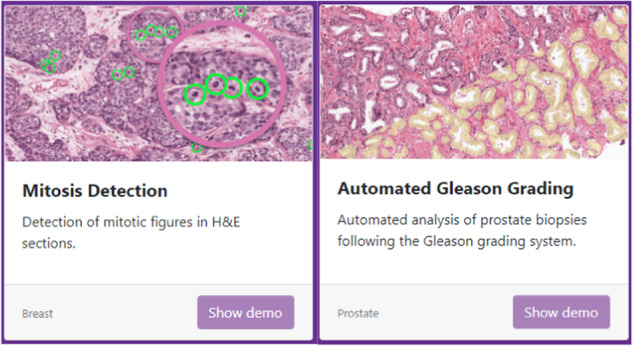
Examples of CPATH algorithms used in introduction interviews.

The online interviews were conducted via Zoom.us V5.6.1 (560) (Zoom Video Communications, Inc., San Jose, CA, USA) or MS teams V1.4.00.8872 (Microsoft, Redmond, WA, USA), based on the preference of the interviewee. Participants provided written informed consent for participation and audio recording prior to the interviews. For participants preferring MS teams, additional verbal consent was given for visual recording. Each interview started with an introduction including a short demo video with audio shared via the “share screen” option in Zoom or MS teams. The video demonstrated the use of one CPath algorithm for mitosis detection and one for prostate biopsy Gleason grading (Fig. 4). Additional information was provided regarding the aim of the study and the interview specifically. After the introduction, the interviewee shared their occupation, experience in pathology, area of focus and experience with both digital pathology and CPath. Participants were then asked about barriers and facilitators regarding CPath on topics related to six of the seven domains of the implementation framework of Flottorp et al, [39]: Innovation factors; Individual professional factors; Professional interactions; Incentives and resources; Capacity for organizational change; and Social, political and legal factors. We did not use the Patient factors domain since pathologists are not in direct contact with patients. Toward the end of the interviews, participants had the opportunity to share relevant thoughts on topics not covered in the interview. The first six interviews were conducted by a PhD student with previous experience both in conducting interviews and focus group discussions, while the remaining interviews were conducted by an MSc student in Biomedical Sciences under the supervision of the PhD student and after receiving a brief interview training as part of the MSc program. The language of the interviews with Dutch pathologists and pathology residents was Dutch. The interviews with pathologists working abroad were only conducted in Dutch when the pathologist was a native Dutch speaker. Otherwise, the interviews were conducted in English. Data was collected until no new information was provided in the interviews on influencing factors. This list of characteristics list included age, years of experience, gender, area of focus within pathology, and type of laboratory. The interviews lasted between 32 and 44 minutes. We used the COREQ checklist to describe the study’s qualitative characteristics [40].

### Data analysis

We analyzed the eSurvey output using descriptive statistics. The interviews were either audiotaped or videotaped in the case of using MS teams and transcribed verbatim for qualitative analysis by ATLAS.ti (version 8.4.20 ATLAS.ti Scientific Software Development GmbH; Berlin, Germany). The transcripts were returned to the respective interviewees for final approval checking for completeness and accuracy. From the accepted transcripts, barriers and facilitators were extracted and coded by two researchers (SE and JS) independently. These codes were then allocated to the domains of the implementation framework of Flottorp et al. [39]. Coding and categorization were discussed until consensus was achieved. A third researcher (RH) was consulted for advice in the event of discrepancy. As a last step, we redefined codes and reorganized coding when needed (i.e. axial coding), resulting in an accurate and concise overview.

## Supplementary information

Supplementary files

## Data Availability

The datasets used and/or analyzed during the current study are available from the corresponding author on reasonable request.
