# Supplementary material for: Pathologists’ first opinions on barriers and facilitators of computational pathology adoption in oncological pathology: an international study
Source: Oncogene. 2023 Aug 16;42(38):2816–27. doi: 10.1038/s41388-023-02797-1 (PMC10504072; doi:10.1038/s41388-023-02797-1)
Supplement: Supplementary file 1 — Supplementary files [file 41388_2023_2797_MOESM1_ESM.pdf]

**Supplementary file 1 Search strategy**

**Search strategy:**

**Histological Techniques [MeSH] OR Pathology [Majr] OR Diagnosis, Computer-Assisted[MeSH] OR Image Interpretation, Computer-Assisted/methods [MeSH] OR Digital pathology[tiab] OR Computational pathology[tiab] OR Whole slide imaging[tiab] OR (digital image analysis[tiab] AND (pathology[tiab] OR histopathology[tiab]))**

**AND**

**Artificial Intelligence [Majr] OR Algorithms [MeSH] OR Neural Networks, Computer [MeSH] OR Artificial intelligence [tiab] OR Algorithm* [tiab] OR Convolutional Neural Network* [tiab]**

**AND**

**Workflow[MeSH] OR policy making[MeSH] OR practice[tiab]**

**AND**

**NOT (Animals[MeSH] NOT Humans[MeSH])**

Applied filters:

- Type of article: Review
- Publication data: 2018 – now

**Supplementary file 2**

**Interview guide**

Thank you very much for your time and effort to participate in this interview! My name is Julie Swillens, researcher at IQ healthcare, Radboud University Medical Center/Sam Engels, master student Biomedical Sciences and intern at IQ healthcare, Radboud University Medical Center. Currently, researchers of the Radboud UMC are working on the development of algorithms applied to digital pathology images. These algorithms could potentially be used for various applications in cancer diagnostics.

To give a short illustration on how these algorithms work, we have made a short demo video. I will now share my screen, so that you can view this video.

However, these algorithms are not yet used in practice to support diagnostics in clinical practice. Thus pathologists and patients cannot yet profit from the potential advantages of the use of these kinds of algorithms by pathologists.

In order to get a better overview of what still needs to happen before we can actually use these algorithms in daily healthcare practice, we have set up this interview study. Our aim is to find out what the opinions are of pathologists and pathology residents regarding the implementation of algorithms in pathology. We will interview pathologists as well as pathology residents, because they are the ones who will actually be able to use these algorithms. They can also inform us about other important stakeholders.

This interview will consist of a number of open questions and a few statements of which we would like to hear whether you agree or disagree.

The interview will take about 45 minutes. There are no right or wrong answers. To not miss any remarks, we would like to record this interview. We have already sent you an informed consent, and I think you have already replied/you can let us know by reply whether you agree with this informed consent. The video will also be recorded automatically, but will be deleted immediately after. Is this acceptable for you?

Are there any questions before we start?

Then I will start the recording right now.

**(1) Could you tell me something about yourself without mentioning your name (for privacy reasons)? (How long have you been working/ what is your focus within pathology)**

**(2) The first questions will be about the potential use of digital pathology and artificial intelligence by pathologists:**

- ***To what extent do you use digital pathology?***
  - *For what applications? (diagnostics, research, education?)*
  - *What is your experience with digital working? Advantages/disadvantages?*
  - *Would you like to make less or more use of digital pathology?*

- ***In what areas do you still experience a lack of knowledge or skills with regard to digital pathology?***
  - *How could this knowledge be improved?*
  - *In what shape or form would you like to acquire more knowledge?*

- ***What do you know about the use of artificial intelligence algorithms in daily pathology practice?***
  - *Where do you experience a lack of knowledge?*
  - *What would you still like to know?*
  - *In what way would you like to obtain more knowledge?*

- ***What is your opinion at this moment about the potential use of artificial intelligence algorithms in daily pathology practice for oncology diagnostics in the near future?***
  - *Why do you think that?*
  - *In what way would you like to use artificial intelligence in daily practice? (leading vs supporting role)*
  - *How do you feel about a national or international guideline about the use of AI in pathology?*
  - *How about the use of AI in 5 years' time?*

- ***What effect do you think the use of artificial intelligence algorithms will have on the diagnostics you do?***
  - *In which way could artificial intelligence algorithms have a positive effect on the diagnostics?*
  - *In which way could artificial intelligence algorithms have a negative effect on the diagnostics?*
  - *Are there other areas which would be improved, except… (already mentioned)? (for example efficiency, quality, etc.)*

- ***How do you think the use of artificial intelligence algorithms will influence the substantive knowledge of pathology?***
  - *Why do you think that?*
  - *In case of negative influence: What could be done to prevent that?*

- ***To what extent do you think you would be able to use artificial intelligence algorithms in practice?***
    - *What could be of help to you in this?*
    - *In case of education: to what extent is additional education needed?*
        - *What kind of education would you prefer?*

***I would now like to present a few statements to you about the use of algorithms within pathological diagnostics. I would like to ask you to tell me whether you agree with them and why.***

- ***<u>I completely trust the output of the algorithm.</u>***
    - *Agree → Why do you agree?*
    - *Disagree → Why do you disagree?*
        - *What is needed to make you trust the algorithm?*

- ***<u>The use of algorithms in daily practice makes pathologists lazy.</u>***
    - *Agree → Why do you agree?*
        - *Why is that a problem? How could it be solved?*
    - *Disagree → Why do you disagree?*

- ***<u>Pathologists in training should not be allowed to learn how to use algorithms during training</u>.***
    - *Agree → Why do you agree?*
        - *When is the right moment to learn how to use them?*
    - *Disagree → Why do you disagree?*
        - *How could this be applied to the training?*

**(3) Now I would like to continue with questions about the algorithm itself:**

- ***In what ways should an algorithm be tested/validated before it can be used in clinical practice? (RCT, prospective, peer-reviewed)***
  - *What do you think is needed for that?*
  - *How should this study be designed? Ex. Minimum amount of images used for testing/ diversity of images, etc.*

- ***What scientific proof is necessary for you before you can use artificial intelligence algorithms in clinical practice?***
  - *What would be the most important outcome measures?*

- ***To what extent should an artificial intelligence algorithm be monitored on whether it actually leads to a better quality of care? (Ex. Less variability between pathologists in grading)***
  - *How should this be monitored?*
  - *Who should monitor this?*

- ***Many scientific articles mention the Black Box when it comes to algorithms. To what extent do you want to know how the algorithm functions exactly before you can use it in practice?***
  - *What would be needed for that?*
  - *In what cases would it be beneficial to know how the algorithm functions?*

- ***At what point in the diagnostic process would you preferably use algorithms, if they were available right now?***
  - *What practical obstacles do you still see for the use of artificial intelligence algorithms in practice?*

***Now I would like to present a few statements to you about the use of algorithms within pathological diagnostics. I would like to ask you to tell me whether you agree and why.***

- ***<u>The use of artificial intelligence algorithms in pathology is the future promise for clinical pathology.</u>***
  - *Why do you think that?*
  - *What do you think is needed for that?*

- ***<u>It is not important who provides the algorithm.</u>***
  - *Why do you think that?*
  - *What role could the provider of the algorithm play in how much you trust an algorithm?*

*In case of **<u>not</u>** having a digital workflow:*

- ***Before we can use artificial intelligence algorithms, first the benefits of a digital workflow need to be further explored.***
    - *What do you think is needed for that?*
    - *How would you estimate the future increase of digital pathology in your lab for the near future?*

*In case of having a digital workflow:*

- ***Algorithms can easily be added to the digital workflow, so that it can function as a CAD (computer aided diagnostics).***
    - *What would the optimal workflow look like?*
    - *Why do you think that?*
    - *What would be needed?*

*When algorithms are already used in clinical practice:*

- *Do you experience any barriers related to the use of the algorithms?*
- *Do you experience any specific barriers related to the software of the algorithms? (for example: very slow, hampering…)*
- *When barriers are experiences, how are these solved? By the lab? By the supplier?*

(4) Then I would now like to present some statements to you about the use of artificial intelligence algorithms and the consequences for the collaboration with other health care professionals:

- ***How will the use (or how does the use, when already used) of algorithms have an impact on the pathologist – clinicians relationship?***

- ***Clinicians can decide whether they want the pathologist to use or not use artificial intelligence algorithms.***

- ***It should be clear for clinicians whether an algorithm was used in making the pathology report.***

- ***Who else plays an important role in the implementation of algorithms in clinical pathology practice?***

Are there any other things that you would like to say about the future implementation of artificial intelligence algorithms in pathology that have not yet been discussed in this interview?

I will now stop the recording.

Thank you very much for your time and effort to participate in our study. You will receive the transcript of the interview for approval.
